# Tissue selecting technique mega-window stapler combined with anal canal epithelial preservation operation for the treatment of severe prolapsed hemorrhoids: A study protocol for a randomized controlled trial

**DOI:** 10.1097/MD.0000000000023122

**Published:** 2020-11-06

**Authors:** Lijiang Ji, Lei Li, Liping Weng, Yuemeng Hu, Hua Huang, Jun Wei

**Affiliations:** a Department of Anorectal Surgery, Changshu Hospital Affiliated to Nanjing University of Chinese Medicine, Changshu, Jiangsu Province; b Tianjin University of Traditional Chinese Medicine, Tianjin, China.

**Keywords:** tissue selecting technique, mega-window stapler, RCT, recurrence, severe prolapsed hemorrhoids

## Abstract

**Introduction:**

Hemorrhoidal disease is one of the most common and frequently occurring benign anorectal disorders, presented with bleeding and prolapsed, and surgery is the main and effective method for severe prolapsed hemorrhoids. Yet, the recurrence rate after procedure for prolapse and hemorrhoids (PPH) is significantly higher. To reduce the recurrence rate and protect the anus function, we try to carry out a randomized, controlled, prospective study to compare the efficacy and recurrence rate of tissue selecting technique (TST) with mega-window stapler (TST-MS) combined with anal canal epithelial preservation operation and PPH combined with external hemorrhoidectomy and inferior internal hemorrhoid ligation operation for the treatment of severe prolapsed hemorrhoids.

**Methods:**

This study is a single-center, evaluator-blinded, randomized, controlled clinical trial. Participants meet the inclusion and exclusion criteria in this RCT will be randomly divided into treatment group (TST-MS combined with anal canal epithelial preservation operation group) and control group (PPH combined with external hemorrhoidectomy and inferior internal hemorrhoid ligation operation) in a 1:1 ratio according to a computer-generated randomization list. The outcomes of recurrence, anal function, intraoperative variables, and postoperative complications will be recorded at different follow-ups.

**Conclusion:**

The findings of the study will help to explore the efficacy and recurrence rate of TST-MS combined with anal canal epithelial preservation operation on the treatment of severe prolapsed hemorrhoids.

**Trial registration:**

This study protocol was registered in open science framework (OSF). (Registration number: DOI 10.17605 / OSF.IO / 4JYNF).

## 1 Introduction

Hemorrhoidal disease is one of the most common and frequently occurring benign anorectal disorders, presented with bleeding and prolapsed. The incidence is extremely high in adults according to different studies.^[1,2]^ In the treatment of hemorrhoids, surgery is still the main method for severe prolapsed hemorrhoids.^[3,4]^ The Milligan-Morgan hemorrhoidectomy (MMH) is the most effective and widely used treatment for symptomatic hemorrhoids of all the conventional hemorrhoidectomy (CH).^[5]^ By removing the hemorrhoid tissue with pathological changes, it could largely damage the anal cushion and the anal canal epithelium, associated with postoperative pain, and a 10% recurrence rate.^[6]^

Since first described by Longo in 1998,^[7]^ procedure for prolapse and hemorrhoids (PPH), also known as stapled hemorrhoidectomy, is an effective alternative by removing the prolapsed rectal mucosa on the dentate line to recover the anatomical position of internal hemorrhoids instead of just removing hemorrhoids. Several studies have shown it is an effective, safe, far less painful, and relative complication free procedure with fewer days off work.^[8–10]^ However, a large number of studies also show that the recurrence rate after PPH is 3.4% to 45%, which is significantly higher than the recurrence rate of conventional hemorrhoidectomy.^[8,11,12]^ Therefore, on the basis of the advantages of PPH technique, it is important to further reduce its postoperative recurrence rate.

Other than that the pathological changes of the hemorrhoids were not removed, the primary reason for the high recurrence after PPH is that for patients with third-grade hemorrhoids with severe internal rectal prolapse, the limited capacity of the PPH stapler cannot remove enough prolapsed tissue.^[13–16]^ At the same time, patients with severe prolapsed hemorrhoids are often accompanied by protruding external hemorrhoids, and approximately 12.7% of patients still suffer from residual external hemorrhoids after PPH stapler surgery.^[17]^ The combined method of PPH technique with the external hemorrhoidectomy and inferior internal hemorrhoid ligation^[18]^ could significantly reduce postoperative edema and residual external hemorrhoids, which expands the indications of PPH technique. However, there is still insufficient tissue removal for severely prolapsed hemorrhoids, which makes internal hemorrhoids prone to prolapse again. Also, external hemorrhoidectomy and inferior internal hemorrhoid ligation could partially damage the dentate line and the anal canal epithelium, which can easily cause stricture of anus and influence the anus function.

To reduce the recurrence rate and protect the anus function, we proposed a novel operation of tissue selecting technique (TST) with mega-window stapler (MS) combined with external hemorrhoid arc resection and suture to preserve the anal canal epithelium for the treatment of severe prolapsed hemorrhoids. The operation could make it better to treat internal hemorrhoids and hemorrhoid mucosa prolapse, reduce the incidence of internal hemorrhoids re-prolapse, completely excise the external hemorrhoids, preserve the anal cushion, dentate line, and anal epithelium, and protect the anal sensation and control bowel control. Therefore, we try to carry out a randomized, controlled, prospective study to compare the efficacy and recurrence rate of TST-MS combined with anal canal epithelial preservation operation and PPH combined with external hemorrhoidectomy and inferior internal hemorrhoid ligation operation for the treatment of severe prolapsed hemorrhoids.

## 2 Method

### 2.1 Study design

**Figure 1:**
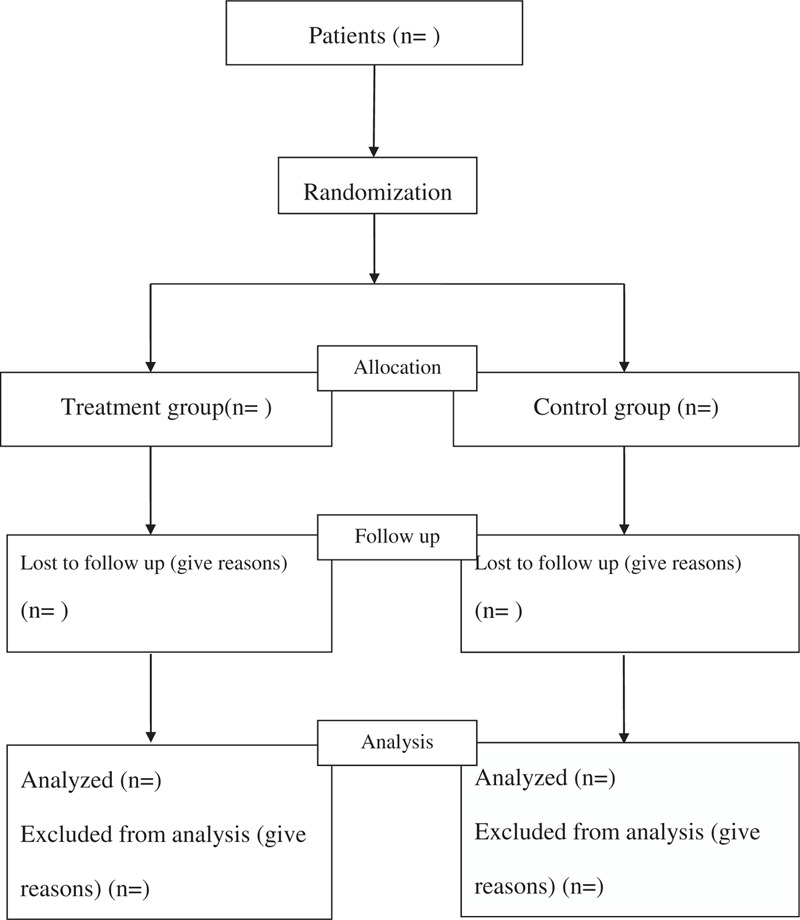
Flow diagram of the study.

This study is a single-center, evaluator-blinded, randomized, controlled clinical trial (RCT). This trial has already been registered in open science framework (OSF) (Registration number: DOI 10.17605 / OSF.IO / 4JYNF). The protocol of the clinical trial has been approved by the Ethics Committee of Changshu Hospital of Traditional Chinese Medicine, and it will be carried out in accordance with the Declaration of Helsinki. The study protocol conforms to the Standard Protocol Recommendations for Interventional Trials (SPIRIT) 2013 Statement,^[19]^ and the results will be reported according to the CONSORT Statement extension for trials^[20]^ (Fig. 1).

### 2.2 Participants

#### 2.2.1 Participants and sample size

Participants meet the inclusion and exclusion criteria in this RCT will be mainly recruited from inpatients of proctology department in Changshu Hospital of Traditional Chinese Medicine.

According to the previous report, the recurrence rate of PPH in the treatment of hemorrhoids was 9.6%. According to the preliminary experiment, the recurrence rate of severe prolapsed hemorrhoids by the TST-MS combined with anal canal epithelial preservation operation was 0%. Taking α = 0.05 and β = 0.2, 85 samples will be needed in each group. Considering the withdrawal rate of 20%, the total number of patients is determined as 204, that is 102 in each group.

#### 2.2.2 Inclusion criteria

Patients are included if they meet the following inclusion criteria:

1. Patients meeting the diagnostic criteria for Circumferential mixed hemorrhoids, with internal hemorrhoids grade III–IV;
2. Patients aged from 18 to 70 years’ old;
3. Patients without obvious deformity of anus before the operation;
4. Written informed consent.

#### 2.2.3 Exclusion criteria

Patients are excluded if they present the following exclusion criteria:

1. Patients who have undergone mixed hemorrhoids surgery, or combined with other operations.
2. Women in pregnancy, lactation, and menstruation.
3. Patients with rectal cancer, rectal polyps, tuberculosis, Crohn disease, and other anorectal diseases.
4. Patients with severe diseases of respiratory, digestive, circulatory, and blood systems, and obvious abnormal liver and kidney functions.
5. Patients with acute inflammatory or thrombotic external hemorrhoids.

#### 2.2.4 Randomization and blinding

The patients will be randomly divided into treatment group (TST-MS combined with anal canal epithelial preservation operation group) and control group (PPH combined with external hemorrhoidectomy and inferior internal hemorrhoid ligation operation) in a 1:1 ratio according to a computer-generated randomization list. Due to the limitation of treatment, the surgeons and patients are not blinded, and the statistical analysis will be carried out by an independent professional researcher who will not know the identification of the groups.

### 2.3 Interventions

The preoperative and postoperative treatment of the 2 groups is the same. Before the operation, the patients will get blood routine test, urine routine test, liver and kidney function, blood glucose, coagulation, infectious disease indicators, electrocardiogram, chest x-ray examination, and abdominal ultrasound, colonoscopy, pathological biopsy, if necessary, to exclude surgical contraindications. The circumference of the anal canal will be measured before the operation. Intraspinal anesthesia will be used in all cases, and the left lateral decubitus will be taken during the operation. After the operation, the total amount of infusion in 6 hours is controlled within 500 mL to reduce the incidence of urinary retention, with 5 consecutive days of routine antibiotics.

#### 2.3.1 Intervention group

1. Internal hemorrhoid surgery will be performed with TST-33-MS (Touchstone International Medical Science Co, Ltd, Suzhou, China) with the following procedure:

① Routinely disinfect the perineum area;
② Appropriately expand the anus, insert an anoscope, and remove the inner tube to reveal the hemorrhoid tissue to be removed;
③ Purse—string suture the hemorrhoid with “0” silk thread 3 to 5 cm from the dentate line only on the mucosa and submucosa;
④ Unscrew the tail wing of the stapler counterclockwise, put the head of the stapler into the anal expander after the head and the body of the stapler are completely loosened, tighten the 2 ends of the purse string around the central rod, lead the suture string out from the symmetrical hole of the stapler through the string lead-out rod, tighten the stapler clockwise, and pull the drawn rectal tissue into the staple groove. At this time, when feel the resistance of the knob, fire the stapler to achieve the cutting and anastomosis. After waiting for 30 seconds, unscrew the tail 3/4 lap counterclockwise and take out the stapler;
⑤ Observe the anastomosis, suture to stop the bleeding if there is active bleeding.

2. Surgery of external hemorrhoids will be performed with the operation of arc resection and suture to preserve the anal canal epithelium with the following procedure:

① After the successful anastomosis, carefully check the remaining external hemorrhoids outside the anus;
② Clamp the 2 ends of the external hemorrhoids with a vascular clamp, and make an arc-shaped incision in the anal canal skin line with a dermatome; peel from the incision and trim the skin edge to smooth if there are severe varicose veins;
③ Perform mattress suture using 3-0 absorbable thread as the skeleton of the incision, and 4-0 absorbable thread for both ends and the suture gap;
④ Examine the resected specimens and submit for pathological examination.

#### 2.3.2 Control group

PPH stapler will be performed for internal hemorrhoids with the same procedure with that in the treatment group; the external hemorrhoids will be stripped to the vicinity of dentate line and ligated at a low position.

### 2.4 Outcome variables

Follow-up of visits to the hospital is required at week 2 and 4 after surgery, and telephone follow-ups are conducted at week 3, 8, and 1 year after the operation, to report and record the following variables (Table 1).

**Table 1:** Outcome variables assessed during the study.

			Post-operation							Follow up			
				
	Preoperation	Intraoperation	12 h	Day 1	Day 2	Day 3	Day 5	Day 7	Wk 2	Wk 3	Wk 4	Wk 8	1 y
Primary outcomes													
Anal function													
Incontinence score	✓										✓		
Circumference of anal canal	✓										✓		
Recurrence										✓	✓	✓	✓
Secondary outcomes													
Operating time		✓											
Intraoperative blood loss		✓											
Weight and volume of excised specimen		✓											
Anal pain			✓	✓	✓	✓	✓						
Bleeding			✓	✓				✓	✓				
Urinary retention			✓	✓	✓	✓	✓	✓	✓				
Anal edge edema			✓	✓				✓	✓				

#### 2.4.1 Primary outcomes

1. Anal function
  ① Incontinence score:
  Wexner score will be used to evaluate the anal function of patients before and 4 weeks after the operation.
  ② Circumference of the anal canal
  Measure the circumference of the anal canal once before and 4 weeks after the operation.
2. Recurrence of internal hemorrhoids and residual external hemorrhoids

Re-prolapse and obvious bleeding of internal hemorrhoids after the operation can be regarded as recurrence. Count the number of recurrences and residual recurrences of external hemorrhoids within 1 year after the operation.

#### 2.4.2 Secondary outcomes

1. Intraoperative variables
  ① Operating time: the time from the beginning of anal expansion to the end of the operation;
  ② Intraoperative blood loss: taking a blooded soaked gauze as 5 mL;
  ③ The weight and volume of the excised rectal mucosa removed by TST-33-MS and PPH.
2. Postoperative complications
  ① Anal pain

The visual analogue scale (VAS) method will be used, and 0 means no pain, 10 means the most pain. The VAS scores will be observed at 12 hours, 1, 2, 3, and 5 days postoperatively. Record the types and frequency of analgesics.

② Bleeding

Observe the bleeding at 12 hours, 1, 7, and 14 days after the operation. Mild bleeding: The bleeding can spontaneously cease; Moderate bleeding: The large bleeding should be stopped after conservative compression; Severe bleeding: The massive bleeding needs suture hemostasis operation when compression is ineffective.

③ Urinary retention

Observe and record the patients receiving at least one catheterization during hospitalization.

④ Anal edge edema

Observe the edema around the anal at 12 hours, 1, 7, and 14 days postoperatively. Grade I: no edema; Grade II: mild edema occupies less than one-fourth of the perianal region; Grade III: edema occupies more than one-fourth and half or more of the perianal area; Grade IV: edema occupies more than half of the perianal area.

#### 2.4.3 Safety evaluation

All possible adverse events (AEs) will be recorded on the Serious Adverse Event Report form, such as bleeding, anal stenosis, anal fissure, pain, urinary retention, anal fistula, prolapse, difficulty in defecation, urgency of stool, pelvic sepsis, systemic complications, and itching. In addition, AEs forms will record all deaths due to any cause during the course of the study.

### 2.5 Statistical methods

Statistical analyses will be performed using the Statistical Package for the Social Sciences (SPSS) v22.0 software (SPSS Inc., Chicago, IL). Statistical testing is 2-sided and *P* < .05 is considered statistically significant. Efficacy outcomes are analyzed on an intent to-treat (ITT) basis who has at least 1 follow-up, and the last observation carried forward rule will be applied to manage missing data. Considering the homogeneity, group T test for normally distributed data or Mann–Whitney *U* test for non-normally distributed data will be used to compare variables between groups. For differences within each group, repeated measured analysis of variance will be used for assessments in different time points, whereas Wilcoxon signed-rank test will be selected for non-normally distributed data.

## 3 Discussion

The single PPH technology for all prolapsed hemorrhoids is no longer in line with current clinical needs, which makes it an important issue to improve the surgical instruments and operation methods, as far as possible to solve the contradiction between curative effect and complications, and to expand the indications. It has become an important problem to be solved in the operation of severe prolapsed hemorrhoids. Therefore, to improve the clinical efficacy reduce the recurrence rate, and protect the anal function for severe prolapsed hemorrhoids, we try to improve PPH technique and expand the indications of stapler to completely preserve the anal cushion and epithelium. Compared with the conventional MMH technique, our novel operation could reduce the damage to the tissues, which will make it faster to heal and cause fewer complications, and compared with current PPH operation, it can significantly reduce postoperatively prolapsed internal hemorrhoids and residual external hemorrhoids, and expand the indications of the stapler, so as to cure hemorrhoids while protecting the anal function.

However, there are still some limitations in this protocol. First, due to the different operation methods, this study cannot be double-blinded, which may lead to certain biases. Second, this study is a single-center study, and the source of patients is relatively single and limited, which may affect the conclusions to a certain extent.

## Author contributions

**Conceptualization:** Liping Weng, Hua Huang.

**Investigation:** Liping Weng, and Lei Li,

**Project administration:** Lijiang Ji, Lei Li, Liping Weng.

**Study design:** Lijiang Ji, Lei Li, and Jun Wei

**Supervision:** Yuemeng Hu, Hua Huang, Jun Wei.

**Validation:** Jun Wei

**Writing – original draft:** Lijiang Ji, Lei Li, Liping Weng.

**Writing – review & editing:** Hua Huang, Jun Wei.
